# Membrane Phase Drives the Assembly of Gold Nanoparticles on Biomimetic Lipid Bilayers

**DOI:** 10.1021/acs.jpcc.1c08914

**Published:** 2022-03-01

**Authors:** Jacopo Cardellini, Lucrezia Caselli, Enrico Lavagna, Sebastian Salassi, Heinz Amenitsch, Martino Calamai, Costanza Montis, Giulia Rossi, Debora Berti

**Affiliations:** † Department of Chemistry “Ugo Schiff” and CSGI, University of Florence, Via della Lastruccia 3, 50019 Sesto Fiorentino, Florence, Italy; ‡ Department of Physics, University of Genoa, Genoa 16146, Italy; ⊥ Institute of Inorganic Chemistry, Graz University of Technology, 8010 Graz, Austria; ∇ European Laboratory for Non-Linear Spectroscopy (LENS), 50019 Sesto Fiorentino, Italy

## Abstract

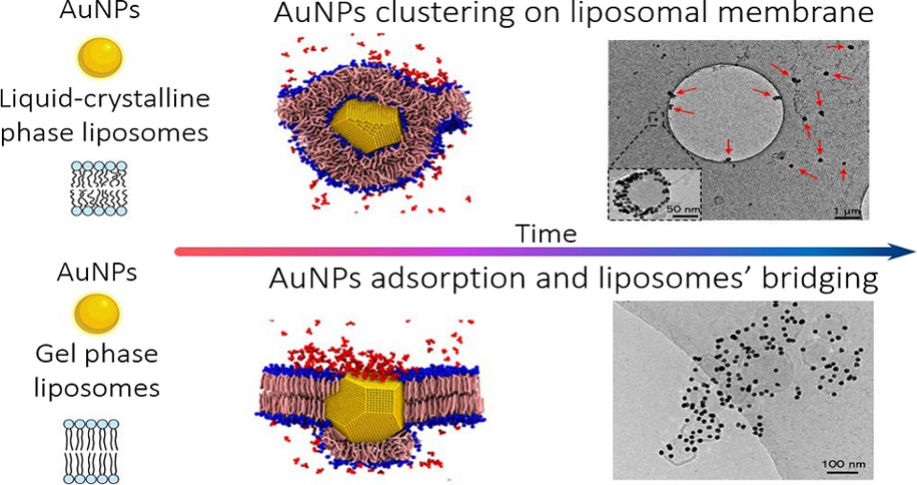

In recent years, many efforts have been devoted to investigating the interaction of nanoparticles (NPs) with lipid biomimetic interfaces, both from a fundamental perspective aimed at understanding relevant phenomena occurring at the nanobio interface and from an application standpoint for the design of novel lipid–nanoparticle hybrid materials. In this area, recent reports have revealed that citrate-capped gold nanoparticles (AuNPs) spontaneously associate with synthetic phospholipid liposomes and, in some cases, self-assemble on the lipid bilayer. However, the mechanistic and kinetic aspects of this phenomenon are not yet completely understood. In this study, we address the kinetics of interaction of citrate-capped AuNP with lipid vesicles of different rigidities (gel-phase rigid membranes on one side and liquid-crystalline-phase soft membranes on the other). The formation of AuNP–lipid vesicle hybrids was monitored over different time and length scales, combining experiments and simulation. The very first AuNP–membrane contact was addressed through molecular dynamics simulations, while the structure, morphology, and physicochemical features of the final colloidal objects were studied through UV–visible spectroscopy, small-angle X-ray scattering, dynamic light scattering, and cryogenic electron microscopy. Our results highlight that the physical state of the membrane triggers a series of events at the colloidal length scale, which regulate the final morphology of the AuNP–lipid vesicle adducts. For lipid vesicles with soft membranes, the hybrids appear as single vesicles decorated by AuNPs, while more rigid membranes lead to flocculation with AuNPs acting as bridges between vesicles. Overall, these results contribute to a mechanistic understanding of the adhesion or self-assembly of AuNPs onto biomimetic membranes, which is relevant for phenomena occurring at the nano–bio interfaces and provide design principles to control the morphology of lipid vesicle–inorganic NP hybrid systems.

## 1 Introduction

The study of interactions between engineered inorganic nanoparticles (NPs) and biomimetic membranes is a very active area of research, inspired by the need to broaden the understanding of the behavior of synthetic nanomaterials at nano–bio interfaces.^1−3^ Over the past several years, the number of reports on the design and application of engineered NPs in nanomedicine has grown exponentially; however, to date the clinical translation of NPs is limited. This limitation is mainly due to a lack of fundamental knowledge on the fate of NPs once in living organisms, which is intimately related to the nature and strength of interactions with biomolecules and biological barriers, such as cell membranes.^4,5^

Specifically, NP adhesion to lipid membranes is a pivotal step that regulates endocytic pathways and biological responses. It involves several interfacial processes, such as ligand–receptor binding,^6^ electrostatic interactions,^7^ and membrane wrapping,^6^ each of which is driven by specific or nonspecific forces.^8^ However, determining the general aspects that govern the nano–bio interaction is not trivial due to the complex and heterogeneous nature of biological membranes. Depending on their composition, biological membranes display different local curvatures, elasticities, and permeabilities, which might affect their response to NP adhesion;^9−11^ moreover, the occurrence of lateral phase-separated domains with different compositions and rigidities (i.e., lipid rafts) is related to cell trafficking phenomena,^12^ suggesting that membrane viscoelastic properties are key to controlling the phenomena at the nano–bio interfaces (from NP adhesion to NP uptake).

The development of synthetic lipid membranes, such as liposomes, giant unilamellar vesicles (GUV), and supported lipid bilayers (SLB), represents a valuable strategy to systematically investigate how such parameters affect the interaction with NPs in simplified and controlled conditions and to predict the relevant aspects that govern the biological fate of NPs.^13−15,2^ In addition, the interaction of lipid membranes with NPs has attracted considerable interest not only for biomimetic studies but also for application purposes. NPs and lipid scaffolds have been successfully combined to form intelligent drug delivery systems (as magnetoliposomes^16,17^ or magnetocubosomes^18,19^) or nanodevices for applications in diagnostics and theranostics.^20,21^ The basic design principles of these devices leverage the spontaneous assembly of NPs with organized lipid assemblies to merge the specific features of inorganic nanoparticles, such as responsivity to magnetic fields^22^ and optical and thermal properties,^23,24^ with the biocompatibility and pharmacokinetic properties of lipid aggregates.^25,26^ The NPs’ response to external forces provides additional control parameters to tune the phase behavior. In addition, the self-organization of lipid assemblies can provide a structural template to control NP–NP interactions and possibly steer the formation of NP suprastructures with enhanced optical or magnetic properties with respect to those of isolated nanoparticles.^19^

One interesting example is the spontaneous association of synthetic unilamellar zwitterionic liposomes and citrate-capped gold nanoparticles (AuNPs).^27−29^ After the spontaneous adsorption of NPs on the lipid membrane, the characteristic localized surface plasmon resonance (LSPR) of AuNPs experiences a red-shift accompanied by the appearance of a secondary peak, centered at about 610 nm, which is associated with a color change of the dispersion from red to purple or dark blue. This optical effect is caused by plasmon–plasmon coupling driven by the decrease of the interparticle distance, indicating the membrane-templated aggregation of NPs. By challenging synthetic liposomes of different rigidities with the citrate-stabilized dispersion of AuNPs, we recently demonstrated that the observed LSPR shifts are closely related to the stiffness of the liposomes, which is determined by the lipidic composition of the bilayer.^30^ In particular, the intensity of the red-shifted peak, i.e., the hallmark of the AuNP aggregation, is minimized for rigid liposomes enveloped by a gel-phase bilayer; in this case, the rigid target membrane is not prone to bend and wrap the NP after NP adhesion, limiting the interfacial NPs–lipid membrane interaction. On the contrary, the lipid membrane–NP interaction and, in turn, the red-shifted peak are maximized for soft liposomes with liquid-crystalline membranes, which are able to efficiently bend and wrap the NPs.^31^

In this work, we address the mechanistic details of this phenomenon by monitoring the interaction of Turkevich–Frens gold nanoparticles with lipid vesicles of different rigidities at different length scales and time scales. Specifically, molecular dynamics (MD) simulations allowed the monitoring of the localized phenomena occurring at the very moment of NP adhesion to the target membranes. Kinetic spectroscopic and scattering data (UV–vis spectroscopy, dynamic light scattering (DLS), and small-angle X-ray scattering (SAXS)) allowed the monitoring the evolution of the NP–lipid vesicle interaction on a colloidal length scale and for longer times (from a few seconds to a few minutes). Finally, cryogenic electron microscopy (Cryo-EM) allowed the determination of the overall morphological characteristics of NP–lipid vesicle hybrids for a long incubation time. Our approach leverages a detailed comprehension of the energetic contributions that drive the formation of hybrid assemblies over different time and length scales, from a few nanometers, where the membrane rigidity plays the major role, to hundreds of nanometers, where colloidal forces govern the interactions. Specifically, we chose two prototypical synthetic liposomal systems (namely 1,2-dioleoyl-*sn*-glycero-3-phosphocholine (DOPC), characterized by a fluid phase at rt.., and 1-dipalmitoyl-sn-glycero-3-phosphocholine (DPPC), characterized by a gel-like phase at r.t.) with markedly different bilayer bending rigidities to show how the first interaction with AuNPs initiates a cascade of colloidal events, which result in completely different hybrid lipid–NP suprastructures.

## 2 Methods

### 2.1 Materials

Tetrachloroauric (III) acid and trisodium citrate dihydrate were provided by Sigma-Aldrich (St. Louis, MO). 1,2-Dioleoyl-*sn*-glycero-3-phosphocholine (DOPC) and 1-dipalmitoyl-*sn*-glycero-3-phosphocholine (DPPC) were provided by Avanti Polar Lipids. All chemicals were used as received. Milli-Q-grade water was used in all preparations.

### 2.2 Synthesis of AuNPs

Anionic gold nanospheres 12 nm in size were synthesized according to the Turkevich–Frens method.^27,32^ Briefly, 20 mL of a 1 mM HAuCl_4_ aqueous solution was brought to the boiling temperature under constant and vigorous magnetic stirring. To the mixture was then added 2 mL of a 1% citric acid solution. The solution was further boiled for 10 min until it acquired a deep red color. The nanoparticle solution was then slowly cooled to room temperature.

### 2.3 Preparation of Lipid Vesicles

To prepare the DOPC and DPPC liposomes, the proper amount of lipid was dissolved in chloroform, and a lipid film was obtained by evaporating the solvent under a stream of nitrogen and overnight vacuum drying. The film was then swollen and suspended in warm (50 °C) Milli-Q water by vigorous vortex mixing to obtain a final 4 mg/ml lipid concentration. The resultant multilamellar vesicles (MVL) in water were subjected to 10 freeze–thaw cycles and extruded 10 times through two stacked polycarbonate membranes with a 100 nm pore size at room temperature to obtain unilamellar vesicles (ULV) with a narrow and reproducible size distribution. The filtration was performed with the Extruder (Lipex Biomembranes, Vancouver, Canada) through Nuclepore membranes.

### 2.4 UV–Vis Spectroscopy

UV–vis spectra were recorded with a Cary 3500 UV–vis spectrophotometer.

### 2.5 Cryo-TEM

On glow-discharged Quantifoil Cu 300 R2/2 grids were applied 3 μL of AuNPs-DOPC and AuNPs-DPPC hybrids. The hybrids were plunge frozen in liquid ethane using an FEI Vitrobot Mark IV (Thermo Fisher Scientific) instrument. Excess liquid was removed by blotting for 1 s (blot force of 1) using filter paper under 100% humidity and 10 °C. Cryo-EM data were collected at the Florence Center for Electron Nanoscopy (FloCEN), University of Florence, on a Glacios (Thermo Fisher Scientific) instrument at 200 kV equipped with a Falcon III detector operated in the counting mode. Images were acquired using EPU software with a physical pixel size of 2.5 Å and a total electron dose of ∼ 50 e^–^/Å^2^ per micrograph.

### 2.6 Small-Angle X-ray Scattering

AuNP–liposome dispersions were studied at the SAXS beamline of synchrotron radiation Elettra (Trieste, Italy), which was operated at 2 GeV and a 300 mA ring current. The experiments were carried out with λ = 1.5 Å, and the SAXS signal was detected with a Pilatus 3 1M detector in the *q*-range from 0.009 to 0.7 Å^–1^. The SAXS curves were recorded in a glass capillary.

### 2.7 Dynamic Light Scattering

DLS measurements at θ = 90° and the ζ-potential determination were performed using a Brookhaven Instrument 90 Plus (Brookhaven, Holtsville, NY). Each measurement was an average of 10 repetitions of 1 min each, and measurements were repeated 10 times. The autocorrelation functions (ACFs) were analyzed through cumulant fitting stopped at the second order for samples characterized by a single monodisperse population, allowing an estimate of the hydrodynamic diameter of particles and the polydispersity index. For polydisperse samples, the experimental ACFs were analyzed through the Laplace inversion according to the CONTIN algorithm. ζ-potentials were obtained from the electrophoretic mobility *u* according to Helmholtz–Smoluchowski equation
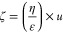
1with η was the viscosity of the medium and ε was the dielectric permittivity of the dispersing medium. The ζ-potential values are reported as averages from 10 measurements.

### 2.8 Gel Electrophoresis

Custom 0.3% agarose gels made with 0.125× Tris–acetate–EDTA (TAE) buffer, i.e., 5 mM Tris (pH 7.6), 2.5 mM acetic acid, and 0.125 mM EDTA, were run in 0.125× TAE buffer using a Bio-Rad submerged horizontal electrophoresis Mini-Sub Cell GT system at 150 V for 10 min.

The external electric field makes the particles migrate according to their electrophoretic mobility, which is expressed by the following equation:
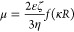
2where *ε* is the dielectric permittivity of the medium, *R* the particle radius, *f*(*κR*) is the Henry function, and ζ is the particle’s ζ-potential.^33^

### 2.9 Computational Methods

#### 2.9.1 Simulation Parameters

All simulations were set up with the coarse-grained Martini force field^34^ and run with Gromacs ver. 2020.6. The cutoff to Van der Waals and electrostatic interactions was set to 1.1 nm, and the dielectric constant was set to ε_r_ = 15. For equilibration and production runs, we set the time step to 20 fs and used the NpT ensemble, with temperature and pressure set to 300 K and 1 bar, respectively. For temperature coupling, we used the velocity rescale thermostat^35^ with τ_T_ = 1 ps. For pressure coupling, we used the Berendsen^36^ barostat in equilibration runs (with τ_p_ = 4 ps) and the Parrinello–Rahman barostat in production runs (with τ_p_ = 12–18 ps), with compressibility set at 3·10^–4^ bar^–1^. We always used the semi-isotropic pressure-coupling scheme.

#### 2.9.2 Au and Citrate Coarse Grained Model

The model for the NPs and citrate was custom developed and described in a previous work.^37^ For Au nanoparticles, we used a 1:1 atom-to-bead mapping scheme, and the Lennard-Jones parameters of the Au–Au interaction were set to the Heinz nonpolarizable potential.^38^ We used two models of Au NPs, both in the shape of a truncated octahedron with diameters of about 8 (12934 beads) and 14 nm (69473 beads). For the 8 nm NP, the size of the largest facets was about 5 nm, comparable to the membrane thickness; for the 14 nm NP, that size was almost doubled (9 nm). The model for citrate was composed of four beads, one representing the hydroxyl terminal group and the others representing the carboxylate terminal groups. Au and citrate non-bonded interactions were parametrized using target properties from atomistic and experimental data, such as the partition coefficient between ether and octanol and adsorption or dimerization free energy profiles.

#### 2.9.3 Simulated systems

We prepared several configurations containing a flat membrane and a citrate-capped Au NP system. At the start of each simulation, the NP was placed in the water phase a few nanometers away from the surface of the bilayer. For the 8 nm NPs, we prepared small boxes (1352 lipids) and larger boxes (5408 lipids) both with DOPC and DPPC; for the 14 nm NPs, we set up a box with 8450 DOPC lipids and another with 8480 DPPC lipids. To obtain gel DPPC, we performed a gelification run of 1 μs using different temperature couplings for water (300 K) and the membrane (250 K), starting from a fluid DPPC membrane.

## 3 Results and Discussion

**Figure 1:**
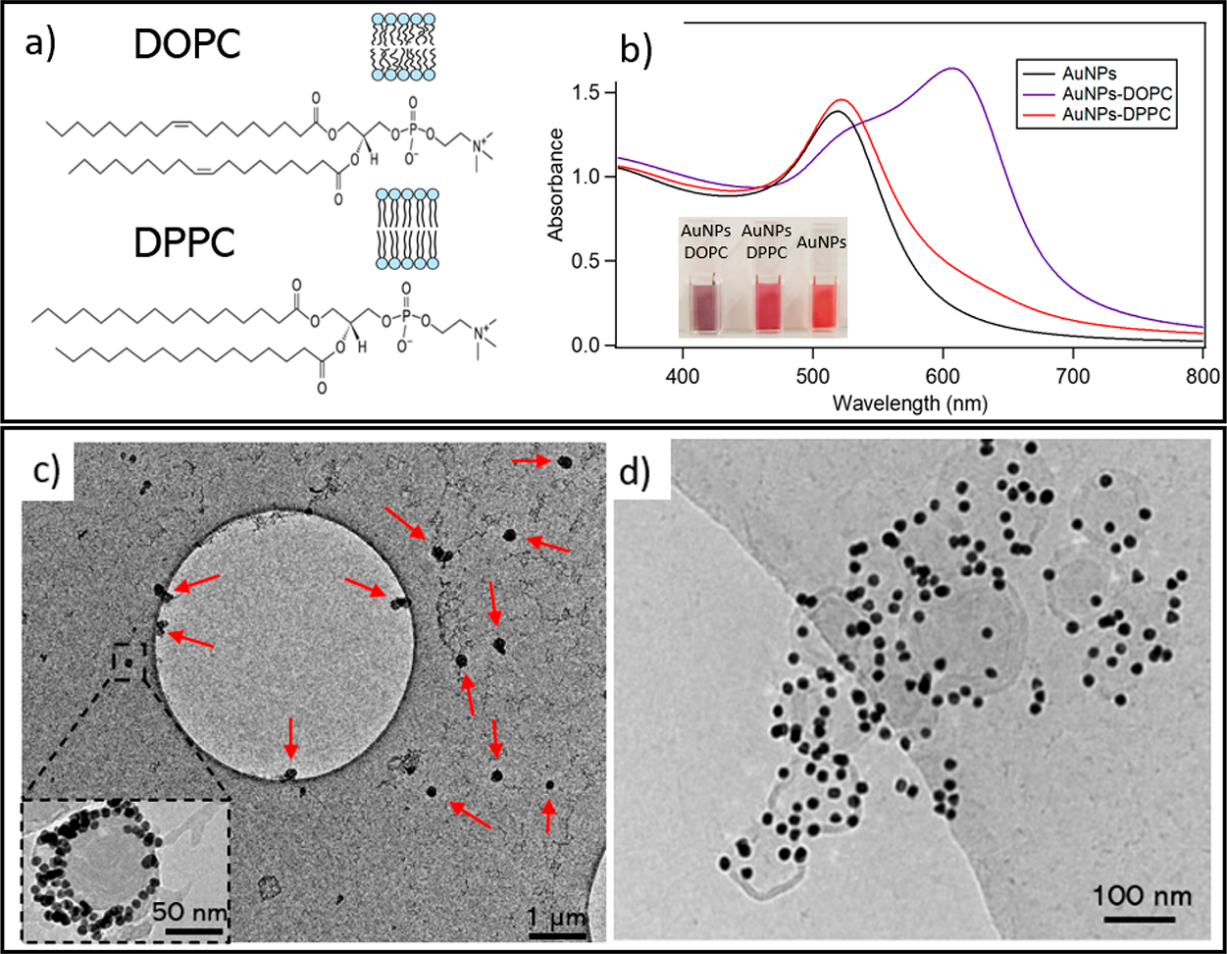
(a) Molecular structures of DOPC (1,2-dioleoyl-*sn*-glycero-3-phosphocholine) with a sketch of the fluid bilayer portion and DPPC (1,2-dipalmitoyl-*sn*-glycero-3-phosphocholine) with a sketch of the gel bilayer portion. (b) UV–visible spectra of AuNPs, AuNPs-DPPC hybrid, and AuNPs-DOPC hybrid collected after 10 min of incubation. Cryo-Tem images of (c) AuNPs-DOPC composites and (d) AuNPs-DPPC composites.

We selected DOPC and DPPC vesicles with a monodisperse size of 100 nm (see the SI) for their well-known difference in terms of membrane stiffness.^39−40^ As sketched in Figure 1 (upper panel), these lipids have the same zwitterionic polar head (PC) but different acyl tails, which are characterized by the absence of unsaturations for DPPC and the presence of two monounsaturated chains (with 6- unsaturations) for DOPC. Such differences dramatically affect the viscoelastic properties of the membrane. At room temperature, DPPC membranes are in a gel state, with highly ordered lipid acyl tails. Conversely, DOPC liposomes are lined by a fluid bilayer with a lower bending rigidity and a higher lateral mobility.^41,42^ As anticipated in the introduction, these structural differences in the lipid acyl chains affect the membrane’s ability to interact with NPs and, in turn, the extent of the membrane-templated clustering of NPs. Accordingly, in a recent work we were able to exploit the extent of AuNP aggregation to estimate the vesicle’s rigidity.^30^

Figure 1b shows some representative UV–vis spectra obtained for 6.3 nM AuNPs with a 12 nm diameter (black curve),^27^ compared to the spectra obtained for NP–vesicles hybrids prepared with a liposome–AuNP ratio equal to 1/16 (red curve for NPs-DPPC, blue curve for NPs-DOPC). This liposome–AuNP number ratio was selected on the basis of our previous publication, which highlights that the aggregation of AuNPs is maximized by low liposome amounts within the mix.

The UV–vis spectra were recorded after 10 min of incubation. As displayed in Figure 1b, the interaction of AuNPs with DOPC and DPPC membranes leads to significant variations in the optical properties of the dispersions. Specifically, the aqueous dispersion of AuNPs-DOPC dramatically turns blue or purple (see the pictures in the inset in Figure 1b). The occurrence of a secondary plasmonic peak at about 610 nm, caused by a decrease in the interparticle distance, reveals AuNPs clustering on the DOPC soft membrane. Conversely, the incubation of AuNPs with rigid DPPC liposomes causes a slight bathochromic shift and a broadening of the characteristic AuNP absorption profile.

To obtain structural information on the AuNP–lipid vesicle hybrid, we performed Cryo-EM imaging. Panels c and d in Figure 1 show representative Cryo-TEM images of DOPC and DPPC vesicles, respectively, challenged with AuNPs. Images were collected after an incubation time of 10 min. The images clearly show that for both liposomes AuNPs adsorb without apparent membrane disruption. However, there is a dramatic morphological difference between these hybrids. Specifically, for fluid DOPC membranes AuNPs adhere to the lipid shell and cluster, in line with the plasmon coupling observed in the UV–vis spectra. In addition, all the lipid vesicles appear as single vesicles, possibly decorated by NP clusters (see red arrows, Figure 1c) without the occurrence of aggregates of the vesicles, giving rise to substantially monodisperse hybrids.

Conversely, for gel vesicles (DPPC, figure 1D) most AuNPs associated the lipid membrane are single particles rather than clusters. Moreover, in all the collected images (see Figures S5 and S6 in the SI for further examples) the DPPC liposomes are connected to each other by AuNP bridges, forming large AuNPs-DPPC vesicle hybrid aggregates.

These results imply that the different degrees of unsaturation of DOPC and DPPC, which lead to the formation of softer or stiffer vesicles, lead not only to different aggregation extents of AuNPs on the lipid membranes but also to the completely different morphologies of AuNP–vesicle hybrids. To understand the mechanistic details of this phenomenon, we monitored this process by combining computational and experimental approaches to access different time scales and length scales

### 3.1 MD Simulations and Molecular Length Scale Characterization of the AuNP–Lipid Membrane Interaction

To gain insights on these lipid phase-dependent interactions at the molecular level and in the very first steps of the AuNP–lipid vesicle interaction, we investigated the very first AuNP–membrane contact using MD simulations. Our simulations rely on a recently developed coarse-grained model of citrate-capped Au NPs.^37^ We have considered two models for the NP. Most of the simulations contain a NP that has a diameter of 8 nm. To rule out significant size effects, we repeated some of the simulations with a larger NP of 14 nm in diameter. Both NPs have the structure of a truncated octahedron, which corresponds to the lowest-energy structure for AuNPs in this size range.^43^ The AuNP citrate coverage in our simulations is 0.97÷1.4 citrate/nm^2^, which is consistent with the available experimental literature.^37^ In ref (37) we calculated the free energy profile for the adsorption of citrate and POPC on the surface of a Au nanoparticle, showing that the interaction with the lipid was thermodynamically favored. Consistently, we observed the spontaneous penetration of Au NPs into POPC fluid bilayers. Here, we ran different sets of unbiased MD simulations in which a single citrate-capped AuNP spontaneously interacts with a DOPC or DPPC lipid bilayer, with the NP starting in the water phase. The list of all simulations and the details of the MD settings are reported in the Methods section.

**Figure 2:**
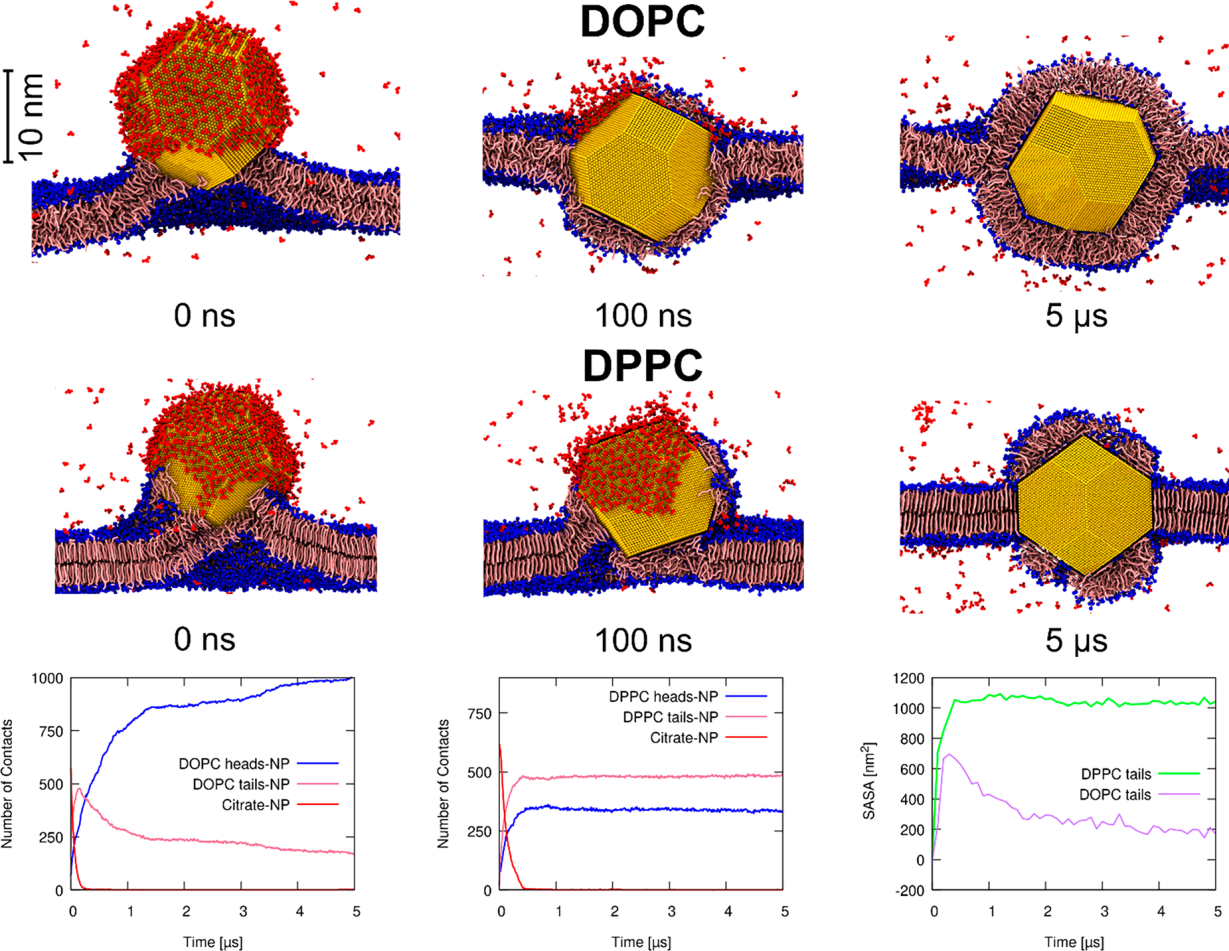
Difference between the penetration process of a citrate AuNP in DOPC and DPPC. For each kind of bilayer, we show three snapshots at significant moments of the wrapping process, where the NP is represented in yellow, citrate is represented in red, the lipid headgroups are represented in blue, and the lipid tails are represented in pink. In the bottom row, we show the time evolution of the number of contacts between the NPs and citrate molecules (in red), lipid headgroups (blue), and lipid tails (pink) for both simulations in addition to the time evolution of the SASA plots of the lipid tails. In the SASA plots, the area is set to 0 for the unperturbed membrane. Time *t* = 0 corresponds to the first NP–membrane contact. The time series are shown only up to 5.0 μs to better highlight the fast initial time evolution, although the simulations reached 10.0 μs in both cases.

In the simulations with the DOPC membrane, the penetration of the NP into the bilayer happened in the first tens of nanoseconds. Similarly to what was described for POPC membranes,^37,44,45^ the membrane wrapped the NP over the following stages (top row of Figure 2): (i) NP–lipid head contact, with the release of citrate in the water phase (ligand exchange); (ii) head–tail flipping of the lipid in contact with the NP; and (iii) the formation of a complete lipid bilayer around the NP, with only heads in contact with its surface. As the figure shows, the head–tail flipping process had already started while the citrate was still being released; overall, the entire process requires less than two microseconds on the coarse-grained time scale of the simulation.

Using similar setups, we performed simulations of a NP interacting with a DPPC bilayer in the gel phase. As a general consideration, we observe that on average the spontaneous onset of the NP–membrane interaction requires more time for NPs-DPPC than for NPs-DOPC, even if the NP starts at the same relative distance from the membrane surface. As the first contact between the Au surface and the lipid headgroups is established, the NP penetrates the DPPC membrane, albeit with significantly different features from the DOPC case. In the central row of Figure 2 we show that the NP pierces the membrane with one of its edges and quickly penetrates the membrane’s hydrophobic core. The NP settles in the membrane with its planar facets in a vertical orientation, parallel to the gel-phase lipid tails. Once the NP reaches the midplane of the membrane, the membrane wrapping of the NP is irregular, forming sparse monolayer patches on the NP surface, and leaving several clusters and stripes of lipids with their hydrophobic tails exposed to the water phase. In this situation, the system dynamics slow dramatically. This significant freezing of the system dynamics can be appreciated and quantified by the plot of the temporal evolution of the NP contacts, as shown in Figure 2 for the 14 nm NP model. The NP contacts fully converge after 1 μs. Furthermore, the plot shows that the initial part of the wrapping process, including the citrate release, is slower than that in DOPC. The gel-phase DPPC lipids adsorbed on the planar NP facets have little to no mobility and do not allow for any further rearrangement of the water-exposed lipid patches. In the plot on the right of the bottom row of Figure 2, we show a time evolution of the solvent accessible surface area (SASA) of the lipid tails. The plot quantifies the water-exposed area of the hydrophobic lipid patches during the interaction of the NP with the two different lipid phases. In DOPC, hydrophobic defects are transient, and their area reaches a maximum during NP penetration and then decreases when a complete DOPC bilayer wraps the NP. On the contrary, in DPPC the hydrophobic defects are permanent on the simulation time scale, as lipid diffusion from the bilayer to the NP surface is almost completely suppressed.

Simulations with 8 nm NPs provide very similar results, as shown in Figure S7 of the SI.

To summarize, molecular dynamics simulations suggest that the outcome of the NP–membrane interaction crucially depends on the membrane lipid phase. Fluid-phase DOPC lipids completely wrap the NP, quickly forming a perfect bilayer around the NP. The external liposome surface would be perturbed little to none by this kind of interaction. On the contrary, the NP interaction with the gel bilayer gets stuck at an intermediate stage of NP penetration. The extremely slow diffusion of lipids in the gel phase freezes the NP–membrane complex in a semiembedded configuration. Neither NP diffusion within the liposome nor further lipid rearrangements would be permitted, leaving significant isolated hydrophobic defects on the surface of the liposome.

### 3.2 Kinetics of Membrane-Templated AuNP Clustering

**Figure 3:**
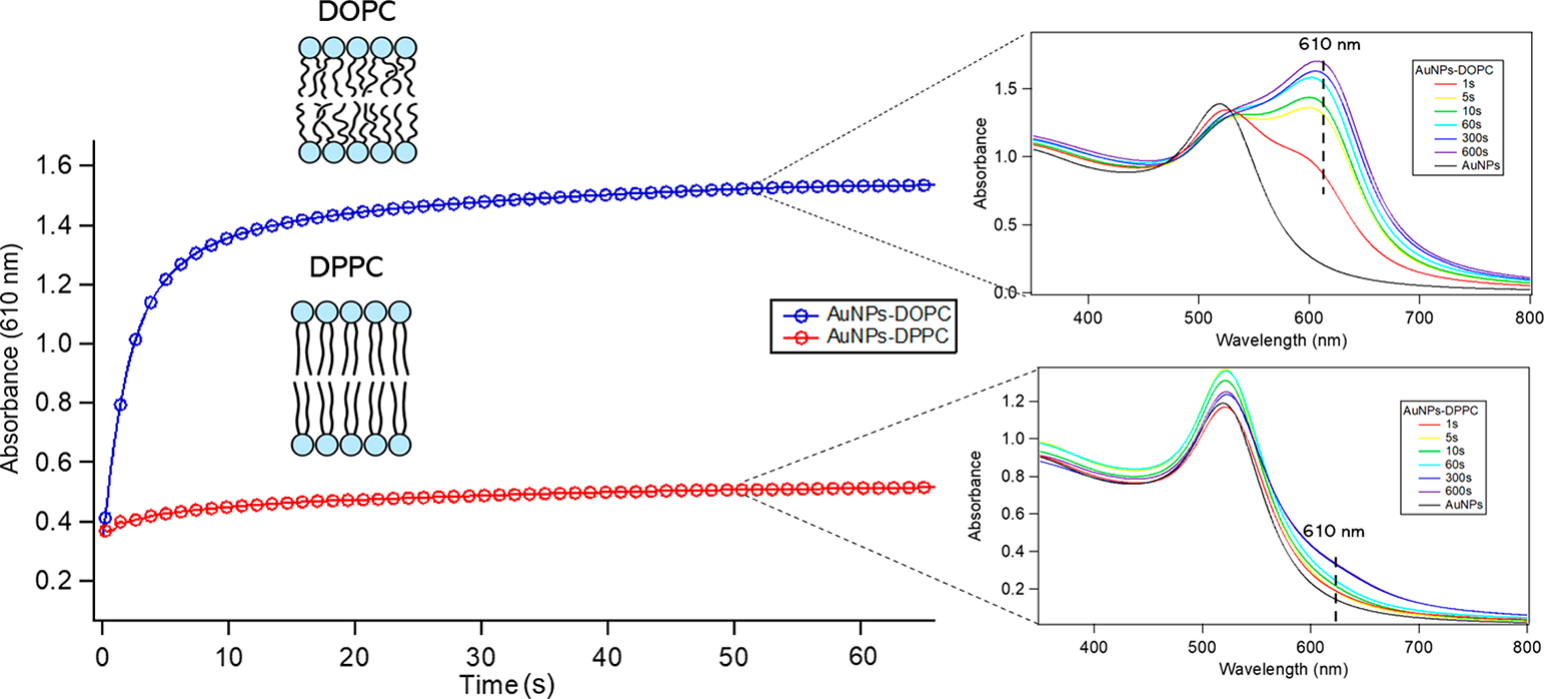
Time evolution of the absorbance of AuNPs-DOPC and AuNPs-DPPC aqueous dispersions (10 μL of 12 nM DOPC or DPPC liposomal dispersions mixed with 300 μL of 6.3 nM AuNPs) at 610 nm.The inset shows UV–visible absorption profiles of AuNPs-DOPC (top) and AuNPs-DPPC (bottom) collected after 1, 5, 10, 60, 300, and 600 s of incubation.

**Figure 4:**
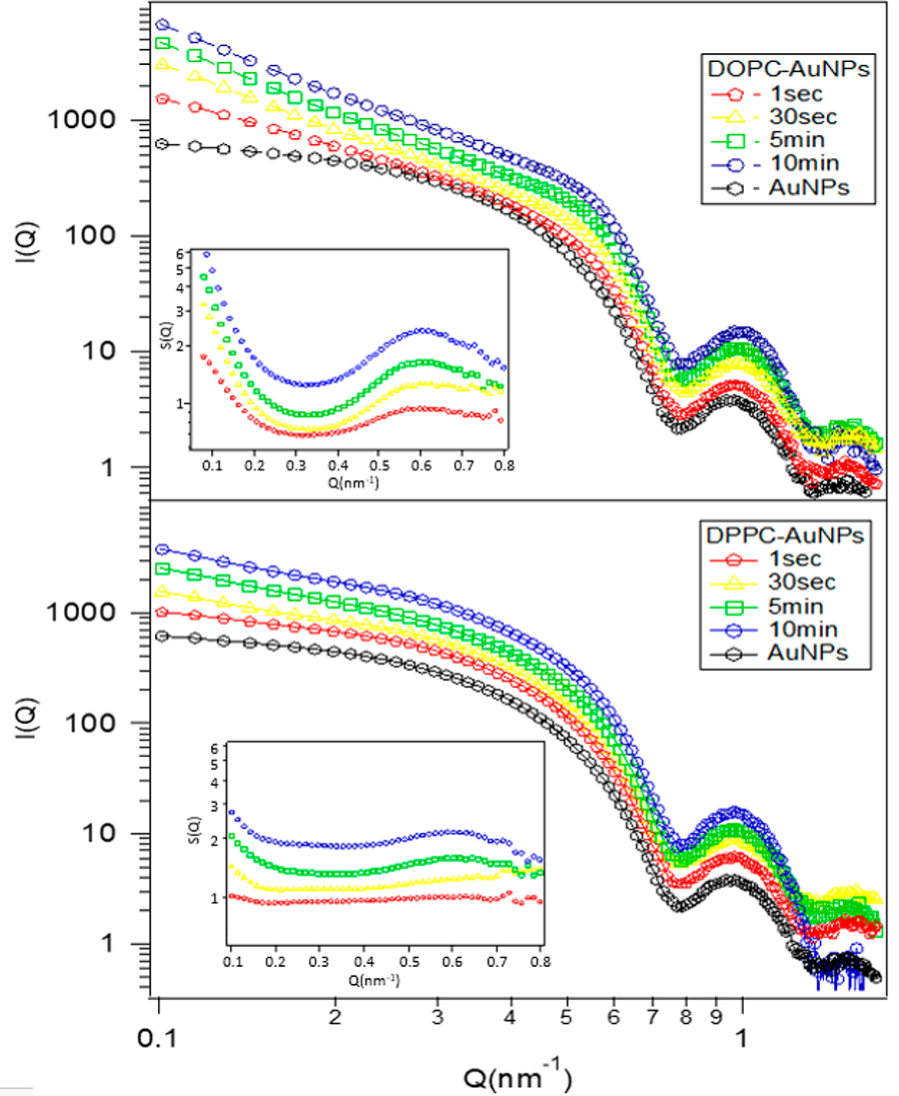
Log–log SAXS profiles of AuNPs-DOPC (top) and AuNPs-DPPC (bottom) hybrids collected after 1 s, 30 s, 5 min, and 10 min of incubation. The inset shows the structure factor of the samples, with correlation peaks related to the center-to-center interparticle distances.

Starting from this very localized and short time scale MD investigation of the adhesion of AuNPs to DOPC and DPPC membranes, we performed tailored complementary experiments to monitor the temporal evolution of AuNP–lipid vesicle hybrids. In particular, we used UV–vis spectroscopy and high-resolution SAXS to monitor the evolution of AuNP adhesion and clustering on the target membrane, following the interaction in the first 10 min of incubation (Figures 3 and 4).

Figure 3 reports the increase over time of the UV–vis absorbance at 610 nm, which is diagnostic for NP clustering,^30^ observed upon the incubation of NPs with DOPC (blue curve) and DPPC liposomes (red curve). The time evolution of the LSPR when AuNPs are mixed with rigid liposomes (DPPC) is characterized by a constant trend, while for lipid vesicles with a soft membrane (DOPC) a relatively sudden absorbance increase occurs that is associated with the appearance of an additional red-shifted signal in the complete spectrum (Figure 3, upper inset). After a few seconds, this shoulder becomes a well-defined secondary plasmonic peak, the signature of plasmon coupling due to the NPs’ close approach. On the other hand, for NPs incubated with DPPC vesicles, the LSPR resonance, peaked at 520 nm for the reference of AuNP dispersion in the same medium, experiences only a very modest absorbance increase at 610 nm. This increase is consistent with the adsorption of NPs onto the liposomal surface, which changes the chemical environment of the NP. In both cases, the absorbance approaches a constant value very rapidly, revealing that NP adhesion to liposomes occurs mostly during the first 30 s of incubation.

The different clustering extent of NPs incubated with DOPC and DPPC liposomes can be interpreted considering the different adhesion modes of NPs, which were previously illustrated for the MD simulations results. Specifically, the membrane’s rigidity modulates its ability to bend around the NPs, resulting in different wrapping extents (full wrapping for DOPC and partial wrapping for DPPC) with a markedly different kinetics of citrate/PC exchange and anion release in the two cases (faster citrate release for the AuNPs-DOPC system and slower citrate release for AuNPs-DPPC system). As hypothesized in a recent study,^31^ this burst of citrate release upon AuNP adhesion on a soft membrane can lead to a local transient increase of the ionic strength. Considering the electrostatic origin of the stability of AuNPs^18^ versus aggregation, this transient ionic strength increase can trigger the aggregation of the neighboring NPs, with resultant clustering on the lipid membrane^33^ in line with UV–vis spectroscopy results. Conversely, for rigid DPPC target membranes the more gradual citrate release strongly limits or completely hampers this effect (see Figures 1 and 3).

We performed high-resolution SAXS at the Elettra synchrotron to determine the structure of AuNP aggregates on the liposomal membrane and to understand the kinetics and mechanism of their formation. The time evolution of the SAXS profiles was followed over the same time frame monitored with UV–vis spectrophotometry. Figure 4 displays the SAXS profiles obtained for AuNPs-DOPC systems (top) and AuNPs-DOPC systems (bottom) for increasing incubation times from 1 s to 10 min. A comparison with the reference scattering profiles of the liposomes and the solvent (water) (see Figure S4 in the SI), highlights that in these experimental conditions the scattering intensity of the vesicles is negligible; therefore, SAXS provides specific information on the evolution of the structural arrangement of AuNPs in AuNP–liposome hybrid systems.

For all the samples, the scattered profile is due to a combination of the following: (i) The AuNPs form factor *P*(*Q*), accounting for the AuNPs’ spherical shape. This contribution is the same in all systems and can be assumed to be equivalent to the SAXS profile obtained for a diluted AuNP dispersion (reported in Figure S1 as a reference). (ii) A structure factor *S*(*Q*), which accounts for the interparticle interactions^46^ such as those that lead to AuNPs clustering on the liposomal surface. To understand the evolution of the organization of AuNPs on the lipid shells, we extracted from each spectrum the structure factor *S*(*Q*), reported in the inset of Figure 3, by dividing the measured scattered intensity of the hybrids by the profile of the AuNPs at the same concentration. The existence of a structure factor different from unity for such a dilute dispersion of AuNPs constitutes to the evidence of a suprastructure of AuNPs. A very coarse-grained analysis provides a correlation between the peak position of *S*(*Q*) and an average distance between particles. For AuNPs-DOPC, the *Q*-position of the peak accounts for an interparticle distance of approximately 11 nm, i.e., particles in direct contact with each other; on the other hand, the weak *S*(*Q*) peak of AuNPs-DPPC suggests a lower degree of positional correlation and a larger average interparticle distance, with only a tiny portion of particles in direct contact. These structural results are consistent with Cryo-EM images (shown in Figure 1), which highlight clusters of AuNPs that are densely packed on the liposomal surface in the case of DOPC and single AuNPs that are separated from each other in the case of DPPC.

The time evolution of the SAXS profiles also provides interesting information on the kinetics of formation of AuNP aggregates on DOPC bilayers. For this hybrid, the *S*(*Q*) peak position is time-invariant, but it becomes more and more defined as time increases. This indicates that AuNPs in the AuNPs-DOPC hybrid are in direct contact with each other since 1 s after mixing. As time progresses, the number of clustered particles present in the aggregates increases; however, the interparticle distance within the aggregates does not change, suggesting a fast and irreversible phenomenon without significant structural rearrangements after the first interparticle contacts. On the contrary, the occurrence of the *S*(*Q*) signal in AuNPs-DPPC system is slower, and the peak is just slightly visible, revealing slower kinetics of the clustering.

In addition, a clear temporal trend is also apparent in the low-*Q* region of the *I*(*Q*) versus *Q* plot for AuNPs-DOPC liposomes. Specifically, we notice a power-law signature that appears as a linear dependence in a double logarithmic plot (see Figure 4, upper panel) with a slope that increases (in absolute value) from −0,.43 ± 0.02 to −2.01 ± 0.02 with time, reaching a constant value after 30 s (−1.90 ± 0.02 after 30 s and −2.00 ± 0.02 after 5 min). This accounts for the dimensionality of the clusters. A −2 slope is consistent with 2D aggregates, while a −1 slope suggests the presence of elongated 1D structures. The observed slope evolution suggests that the final shape of the AuNP aggregates is a 2D cluster, in line with a full coverage of the liposomal surface by AuNPs (as hypothesized in a recent study^31^). Moreover, it appears that overall the AuNP clusters evolve in dimension, with a progression from a 1D aggregate to a 2D aggregate (see Table S5). Such a rapid variation of the power-law exponent highlights that NP aggregation is a fast process that requires only a few tens of seconds of incubation to approach an equilibrium arrangement for AuNPs, in line with the time-evolution of the LSPR monitored through UV–vis spectroscopy (Figure 3). A slight slope increase (in absolute value) in the low-*Q* region can also be noticed for the AuNPs-DPPC hybrids (from −0.43 to −1.02). This variation accounts the absorption and partial aggregation of AuNPs on the DPPC shells, forming much less densely packed AuNPs clusters.

Overall, SAXS and UV–visible measurements are consistent with the hypothesis that the rigidity of the bilayer controls the extent of AuNP clustering. However, in both cases (DOPC and DPPC) the arrangement of AuNPs reaches a stable configuration very rapidly.

**Figure 5:**
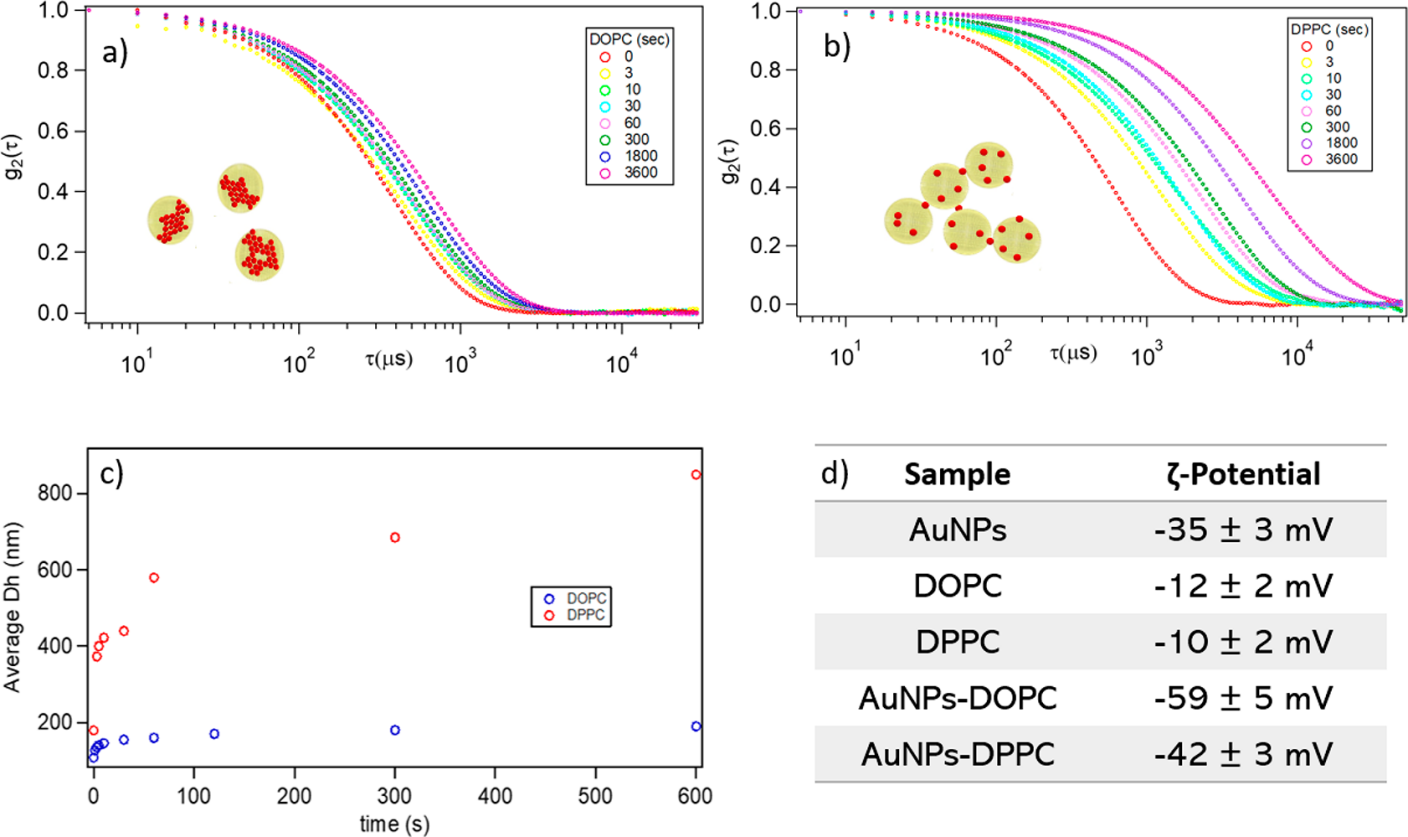
10 μL of 12 nM DOPC or DPPC liposomal dispersions was mixed with 300 μL of 6.3 nM AuNPs and the hydrodynamic dimension of the hybrids were followed up to one hour. (a) Time evolution of the DLS curves of the AuNPs-DOPC hybrid. (b) Time evolution of the DLS curves of the AuNPs-DPPC hybrid. (c) Time evolution of the average hydrodynamic diameter evaluated by the DLS curves for AuNPs-DOPC and AuNPs-DPPC composites. (d) ζ-Potentials of citrate AuNPs, pure DOPC and DPPC vesicles, and AuNPs-DOPC and AuNPs-DPPC hybrids.

We then monitored the hybrids at the colloidal length scale, specifically addressing their colloidal stability with DLS measurements for longer incubation times (up to one hour). The main results are summarized in Figure 5, with details on preparation and data analysis reported in the caption.

Panels a and b in Figure 5 report representative normalized DLS curves obtained for DOPC-AuNPs systems and DPPC-AuNPs systems, respectively, within 1 h of incubation. The average diameters of AuNPs-–vesicle hybrids for increasing incubation times (Figure 5c) were determined by analyzing the decay times of the autocorrelation functions through a cumulant fitting stopped at the second order. The hydrodynamic diameters (*D*_h_) of DOPC vesicles and AuNPs show an increasing trend over time upon interaction. In a few minutes, a hybrid system with a *D*_h_ of about 180 nm forms, which is consistent with the size of the lipid vesicles surrounded by a shell of inorganic nanoparticles.

On the other hand, the interaction of AuNPs with DPPC membranes leads to a sharp increase in the decay times of the autocorrelation functions, i.e., of the sizes of the hybrids. Despite SAXS and UV–vis point toward a weaker interaction of AuNPs with rigid liposomes, DLS results highlight a dramatic decrease of their colloidal stability, with the relatively fast formation and flocculation of micrometer-sized aggregates and, eventually, precipitation. This result is consistent with the aggregation of vesicles, with AuNPs acting as bridging agents in the case of DPPC (see Cryo-EM images in Figure 1 and the insets in Figure 5 a and b).

To further address such a dramatic difference in the colloidal stability of the AuNPs-DOPC and AuNPs-DPPC hybrids, we also measured the ζ-potential (Figure 5d). The ζ-potential is a quantitative measure of the kinetic colloidal stability^47^ of the hybrids. DOPC and DPPC vesicles have a slightly negative ζ-potential in water, in agreement with the literature,^48^ while hybrid systems display an increase in absolute values toward more negative potentials. In particular, the electrostatic stabilization is very pronounced for the DOPC hybrids and may explain their colloidal stability. Conversely, for DPPC the increase (in absolute value) of the ζ-potential upon the adhesion of AuNPs is significantly lower, suggesting that an overall lower surface charge of AuNPs-DPPC hybrids might be related to a higher colloidal instability.

Gradzielsky et al. have shown that the liposome decoration by anionic inorganic particles causes an increase of the absolute value of the surface ζ-potential due to the increase of electric charges on the lipid shell, resulting in the formation of metastable hybrid nano-objects.^49^ The colloidal stabilization was reached only due to the absorption of a sufficient number of particles. Conversely, a low number of particles cannot prevent the liposome fusion and ensure colloidal stability, leading to the rapid destabilization of the dispersion.

In the present case, we hypothesize that the high number of AuNPs assembled on the DOPC liposomes determines a local increase of the negative charge, stabilizing the colloidal dispersion through electrostatic repulsion. For DPPC vesicles, given the lower number of adsorbed NPs on membrane, the electrostatic repulsion is not sufficient to overcome attractive interactions, leading to liposome bridging and flocculation. The dependence of the liposome’s stabilization on the extent of surface coverage by charged nanoparticles is well-known in literature;^50−53,26^ however, in the present case we cannot rule out that the phase of the lipid membrane may also play a role.

Specifically, as shown in the MD simulation, considering that the AuNPs adsorbed on rigid membranes in a semi-embedded state are partially exposed to the external aqueous solution, the attractive interaction with the neighboring vesicles may be favored. Moreover, a hydrophobic interaction between the hydrophobic patches exposed to the water in the AuNPs-DPPC semi-embedded configuration might also have a prominent role, eventually leading to the flocculation of the hybrids.

Considering all these results, two different interaction mechanisms can be considered depending on the vesicle’s rigidity, starting from the first particle–bilayer contact up to the colloidal regime.

**Figure 6:**
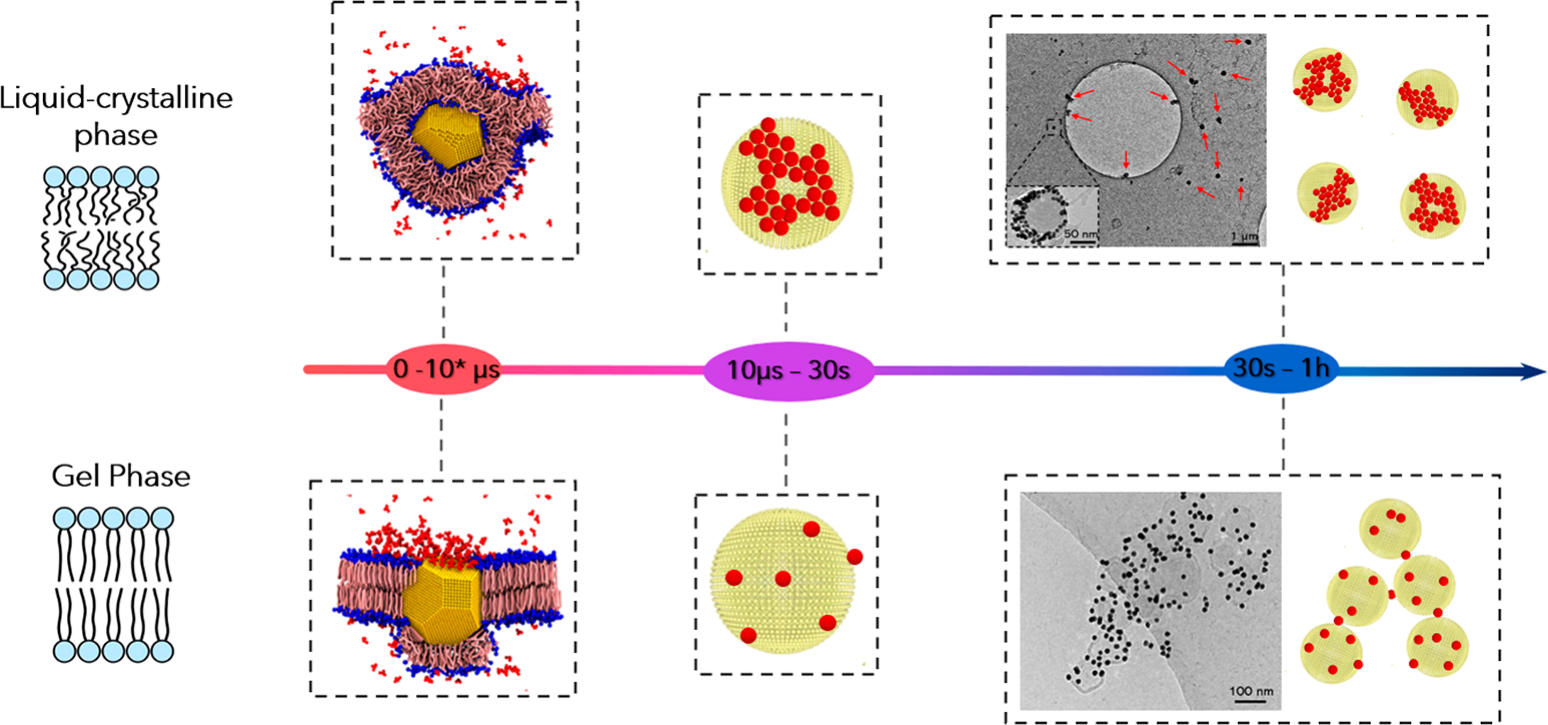
Schematic representation of the interaction mechanisms dependent on the vesicle rigidity. First, the adhesion of the particles and the citrate release occur. Second, the AuNPs in the proximity of the interaction site aggregate according to the kinetics of the citrate release. Finally, the hybrids evolve in single vesicles decorated by AuNPs or in flocculated objects where the particles act as a bridge. We remark that the simulated time scale (10 μs) corresponds to 10–100 μs in real time due to the acceleration of the dynamics that is intrinsic to the use of a coarse-grained model.

The schematic representation of the processes is reported in Figure 6. (i) First, a AuNP adheres on the lipid membrane, undergoing a membrane-wrapping phenomenon; the extent of AuNP wrapping by the membrane depends on the stiffness of the vesicle, which in turn depends on the state of its membrane, either the gel phase (rigid) or the liquid-crystalline phase (soft). (ii) The first step also controls the kinetics of citrate release upon AuNP adhesion to the target membrane, which is determinant in controlling the interaction of the AuNPs adhered on the liposomal surface with neighboring particles. This in turn leads to the formation of AuNP clusters (on soft membranes) or the separate adhesion of AuNPs (on rigid vesicles). (iii) The different amounts of adhered particles (tight clusters or single particles) and their different natures (protruding from the liposomal surface or wrapped by the lipid membrane) affect the colloidal stability of AuNP–lipid vesicle hybrids, leading to the formation of AuNP-decorated vesicles in the case of soft vesicles and extended vesicles clusters bridged by AuNPs in the case of rigid vesicles.

### 3.3 Proof of Concept: Separation of Biomimetic Vesicles of Different Stiffnesses

The whole set of experimental measurements that were performed allowed us to discriminate between two interaction mechanisms, which were determined by the composition of the vesicles, i.e., on their membrane rigidity. Such a physicochemical characterization can not only be instrumental in fundamental studies but can also provide a design principle to build novel hybrid materials with the controlled clustering of both NPs and lipid vesicles and, possibly, different tailored colloidal and functional properties dependent on the rigidity of the lipid vesicles.

**Figure 7:**
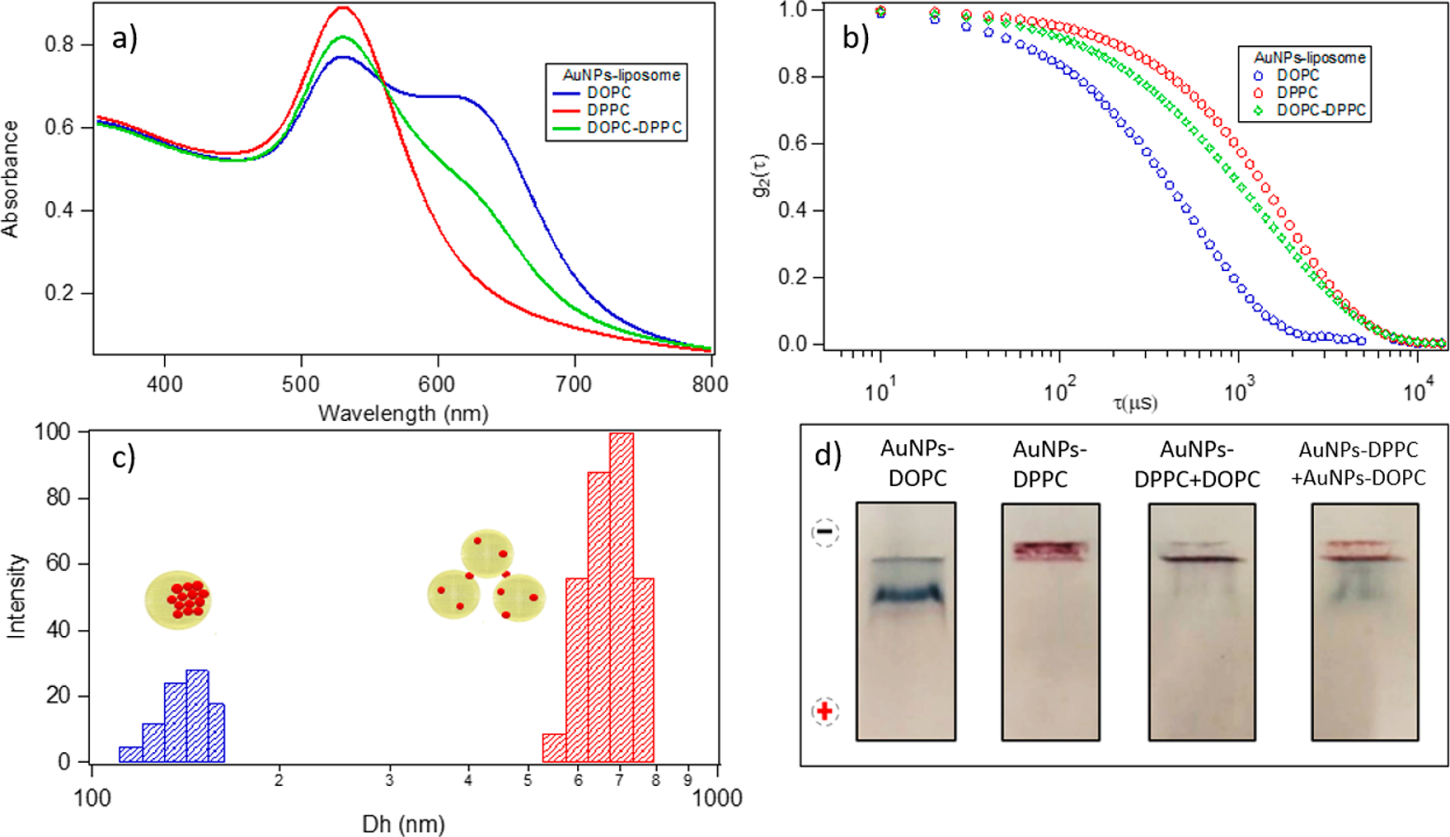
AuNPs-DOPC and AuNPs-DPPC hybrids were prepared mixing 10 μL of 12 nM liposomal dispersions with 300 μL of 6.3 nM AuNPs. To prepare the AuNPs-DOPC+DPPC sample, 300 μL of a 6.3 nM aqueous solution of the AuNPs was added to a mixture of 5 μL of the 12 nM DOPC dispersion and 5 μL of the 12 nM DPPC dispersion. (a) UV–visible spectra of AuNPs-DOPC, AuNPs-DPPC, and AuNPs-DOPC+DPPC samples. (b) DLS curves of AuNPs-DOPC, AuNPs-DPPC, and AuNPs-DOPC+DPPC sample. (c) Double population obtained by the DLS measurement of the AuNPs-DOPC+DPPC composites. (d) Agarose gel electrophoresis analysis of the samples (from left to right: AuNPs-DOPC, AuNPs-DPPC, AuNPs-(DOPC+DPPC), and AuNPs-DOPC+AuNPs-DPPC).

In addition, the different sizes and ζ-potentials of hybrid AuNP–lipid vesicle systems for rigid (DPPC) and soft (DOPC) vesicles can be exploited as a separative strategy for dispersions of biogenic vesicles (EVs) of different rigidities, given that the purification and separation of extracellular vesicles is still an open issue.^54^ In the following, we will illustrate a proof-of-concept experiment for the separation of DOPC and DPPC vesicles through incubation with AuNPs, followed by gel electrophoresis (Figure 7). To this aim, we prepared a sample containing both DOPC and DPPC and monitored the interaction with AuNPs via UV–visible spectrometry and DLS measurements over 5 min (Figure 7).

The intensity of the red-shifted peak of the mixed sample is intermediate between those obtained for the DOPC and DPPC samples. This behavior suggests that a portion of the AuNPs clusters on the liquid-crystalline membranes, while the remaining ones adhere to the gel-phase vesicles and initiate the bridging process.

Concerning DLS measurements, the correlation function (Figure 7b) of the AuNPs-DOPC+DPPC hybrid clearly displays two main decay times, suggesting the presence of two populations, one consistent with the size of the AuNPs-DOPC hybrid and the other with a larger and less colloidally stable population of the AuNPs-DPPC hybrid. The smaller population peaked at around 160 nm (Figure 7c), in line with the size of the lipid vesicles of DOPC with a shell of aggregated AuNPs, and the second one, which was much more polydisperse, was centered at 600 nm, consistent with the presence of bridged DPPC liposomes.

These results show that in dispersions where DOPC and DPPC vesicles are present the overall interaction of AuNPs is a combination of the two mechanisms (decoration of single vesicles with AuNP clusters for DOPC liposomes and the adhesion of single AuNPs on vesicles and subsequent bridging for DPPC liposomes); therefore, once the mixed liposomal dispersion, whose liposomes are characterized by practically identical sizes and surface charges, is incubated with AuNPs, the mixture evolves in a combination of highly charged single vesicles decorated by AuNPs (for DOPC vesicles) and less-charged extended aggregates bridged by AuNPs. After interacting with AuNPs, AuNPs-DOPC and AuNPs-DPPC hybrids possess different ζ-potential values and sizes, making the two hybrids easily separable. To prove this hypothesis, the mixed dispersion was analyzed through agarose gel electrophoresis (AGE). This technique has found multiple applications in the field of colloid and NP characterization to determine the charge and size variations of a dispersed system, allowing the separation of two colloidal populations as a function of their differences in dimension and surface potentials.^33^

We prepared an agarose gel with four wells (reported in Figure 7d) to compare the electrophoretic mobility of the mixed samples with respect to the isolated samples of AuNPs-DPPC and AuNPs-DOPC: (1) DOPC-AuNPs hybrids, (2) DPPC-AuNPs hybrids, (3) AuNPs added to the DOPC–DPPC mixture, and (4) a mixture of AuNPs-DOPC and AuNPs-DPPC hybrids (hybrids mixed after their formation) (Figure 7d from left to right: AuNPs-DOPC, AuNPs-DPPC, AuNPs-(DOPC+DPPC), and AuNPs-DOPC+AuNPs-DPPC). Immediately prior to loading in the well, 15 μL of each sample was mixed with 5 μL of glycerol to improve the sample deposition.

In the first well, practically all the objects migrated from the starting position toward the end of the channel after ten minutes. Conversely, the AuNPs-DPPC hybrids in the second well did not move from the deposition point. These differences in electrophoretic mobility are primarily due to the increase of the hydrodynamic diameter, since this sample is composed of micrometer-sized bridged objects (see the Materials and Methods section for the gel preparation).

When both DOPC and DPPC liposomes are present (i.e., 3 and 4), gel electrophoresis detects two different populations, one with an electrophoretic mobility very similar to that of DOPC-AuNPs and one with a population that behaves similar to the AuNPs-DPPC hybrid.

Even if very preliminary, this experiment shows that the self-assembly of AuNPs on lipid vesicles, which is highly dependent on the rigidity of the lipid vesicles, can be exploited to separate vesicles of similar surface charge and size based on their rigidity. Overall, this proof-of-principle paves the way to explore several possible applications, such as a semiquantitative assay of the rigidity of membrane-enveloped nano-objects in complex samples of biological or synthetic origin or the real-time monitoring of complex phenomena such as liposome fusion, lipid exchange between liposomes, and transient raft formation.

## 4 Conclusions

Understanding the interaction of inorganic nanoparticles with synthetic vesicles is crucial both to improve our fundamental knowledge of the main determinants driving phenomena at the nano–bio interface and to inspire novel design principles to build functional hybrid nanomaterials for biomedical applications. In this study we have addressed the interaction of citrate-capped AuNPs with synthetic liposomes of different rigidities over different length and time scales. We showed that the adhesion pathway of AuNPs to the target membrane is governed at the molecular level by the physical properties of the lipid bilayer (i.e., its rigidity). This different initial and local interaction mechanism results, for long incubation times and at the colloidal length scale, in dramatic differences in the morphologies, structures, and physicochemical properties of AuNP–liposomes hybrids. Overall, we highlight the multiscale nature of the formation and the properties of AuNP–vesicles hybrids. Depending on the physical state of the bilayer, the energetic balance of NP wrapping is different. This delicate energetic balance, which concerns a phenomenon at the nanometer scale, initiates a cascade of events that regulate the colloidal interactions up to the micrometer scale and control the final morphology of the hybrids, which range from single soft vesicles decorated by AuNP clusters to flocculated rigid liposomes bridged by single AuNPs. In addition, some preliminary results demonstrate the possibility to exploit this interaction cascade to separate mixtures of chemically and colloidally identical vesicles based on their membrane rigidity.This mechanistic understanding paves the way to engineer and finely control hybrids where soft and biocompatible vesicles are combined with the hard properties of citrate-stabilized inorganic NPs through simple self-assembly.

## Supporting Information Available

Supplementary SAXS, UV–vis, and cryo-EM data (PDF)
