# Supplementary material for: Membrane Phase Drives the Assembly of Gold Nanoparticles on Biomimetic Lipid Bilayers
Source: J Phys Chem C Nanomater Interfaces. 2022 Mar 1;126(9):4483–94. doi: 10.1021/acs.jpcc.1c08914 (PMC8919252; doi:10.1021/acs.jpcc.1c08914)
Supplement: Supplementary file 1 — jp1c08914_si_001.pdf [file jp1c08914_si_001.pdf]

Supporting Information for:

Membrane Phase Drives the Assembly of Gold Nanoparticles on Biomimetic Lipid Bilayers

Jacopo Cardellini[a], Lucrezia Caselli[a], Enrico Lavagna[b], Sebastian Salassi[b], Heinz Amenitsch[c], Martino Calamai[d], Costanza Montis[a], Giulia Rossi[b], Debora Berti[a]*

[a] Department of Chemistry "Ugo Schiff" and CSGI, University of Florence, via della Lastruccia 3, 50019 Sesto Fiorentino, Florence, Italy;

[b] Department of Physics, University of Genoa, Genoa 16146, Italy;

[c] Institute of Inorganic Chemistry, Graz University of Technology, 8010 Graz, Austria;

[d] European Laboratory for Non-linear Spectroscopy (LENS), 50019 Sesto Fiorentino, Italy;

	Page
**Supplementary Characterization of Gold Nanoparticles**	**S2**
Small Angle X-Ray Scattering	*S2*
Dynamic Light Scattering and Z-Potential	*S3*
UV-Vis Spectroscopy	*S4*
**Supplementary Characterization of Liposomes**	**S5**
Dynamic Light Scattering and Z-Potential	*S6*
Evaluation of liposomes concentration	*S6*
Small Angle X-Ray Scattering	*S7*
**Supplementary Characterization of liposomes/AuNPs hybrids**	**S8**
Preparation of liposomes/AuNPs hybrids	*S8*
Cryo-TEM	*S9*
MD simulations	*S10*
Small-Angle X-Ray Scattering	*S10*

Agarose gel electrophoresis	*S13*
**Bibliography**	**S14**

Supplementary Characterization of Gold Nanoparticles

Small Angle X-ray Scattering

SAXS measurements on AuNPs aqueous dispersion were carried out in sealed glass capillaries of 1,5 mm diameter.

The structural parameters (Table S1) of citrated gold nanoparticles were evaluated from the SAXS profile of their diluted water dispersion (Figure S1), according to a spherical form factor and a Schulz size distribution. In this concentration range, we can safely assume that there are no interparticle interactions are present, and that the structure factor S(Q) equals in the whole range of scattering vectors. Thus, the scattering profile of the particles derives from their form factor, P(Q). The SAXS spectrum reported in Figure S1 is fully consistent with the characteristic P(Q) of spherical particles with an average diameter of about 5.8 nm. The clear presence of P(Q) oscillations in the high Q

region is consistent with a relatively low polydispersity of the synthesized AuNPs.

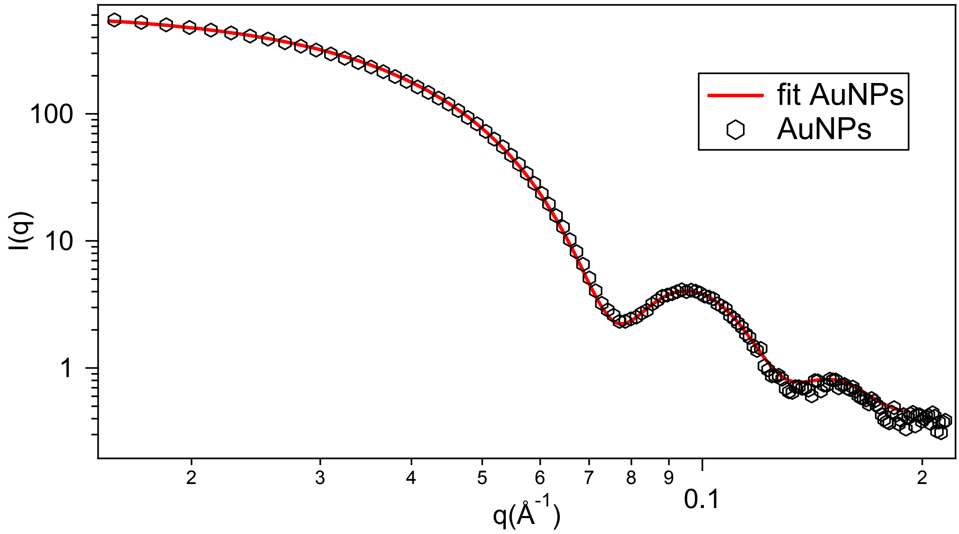

Figure S1 *Experimental SAXS curve (red markers) obtained for AuNPs and curve fit (solid black line) according to the Schulz spheres model from the analysis software package SasView. The size and polydispersity obtained from the fitting procedure are summarized in the Table S1 below.*

	R_{core} (nm)	poly
**AuNP**	5,78	0,095

Table S1 *Structural parameters of the nanoparticles obtained from the analysis of SAXS curves according to the Schulz spheres model.*

Dynamic Light Scattering and Z-Potential

AuNPs hydrodynamic diameter and surface charge in MilliQ water were evaluated through Dynamic Light Scattering and Zeta-Potential, respectively, and reported in Table S2.

	D_h (nm)	Z-Potential (mV)
**AuNPs**	20 ± 0,6	-35 ± 3

Table S2 *Hydrodynamic diameter obtained from Dynamic Light Scattering and Zeta Potential values of AuNPs.*

UV-vis Spectroscopy

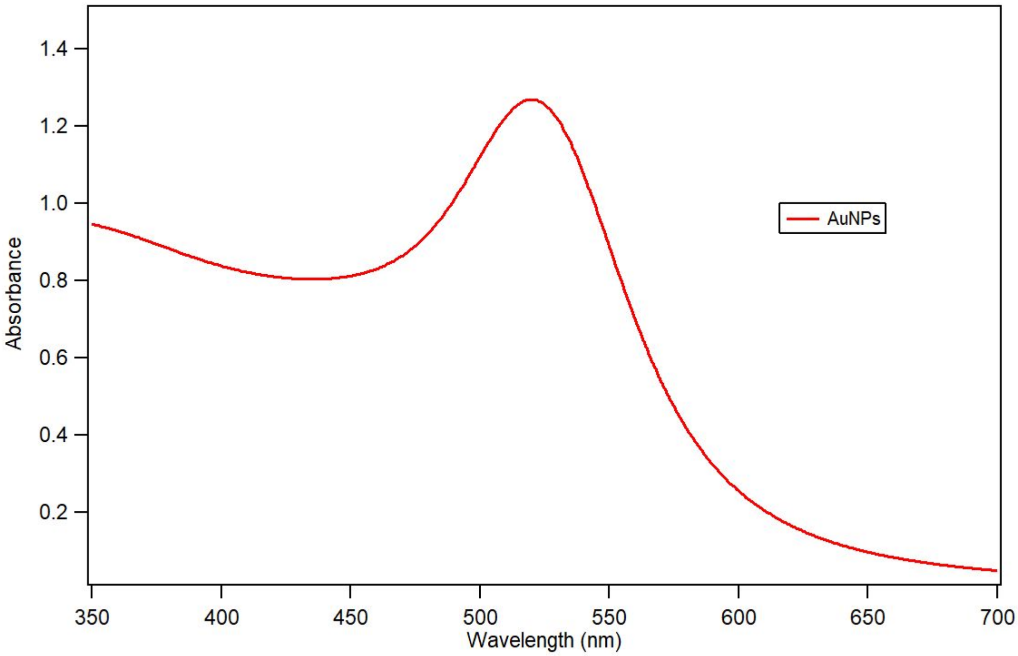

Figure S3 *UV-Vis absorption spectra of AuNPs after 1:3 dilution in water. The plasmon absorption peak is at around 520 nm.*

To further evaluate the AuNPs size through UV-Vis spectroscopy we exploited the following equation[1]:

$$d = \exp\left(B_1 \frac{A_{spr}}{A_{450}} - B_2\right)$$

with d diameter of gold nanoparticles, A_{spr} absorbance at the surface plasma resonance peak, A_{450} absorbance at the wavelength of 450 nm and B_1 and B_2 are dimensionless parameters, taken as 3 and 2,2, respectively. The diameter value obtained is of 12,3 nm.

The concentration of citrated gold nanoparticles was determined via UV-Vis spectrometry, using the Lambert-Beer law ($E(\lambda) = \varepsilon(\lambda)lc$), taking the extinction values $E(\lambda)$ at the LSPR maximum, i.e. λ = 521 nm. The extinction coefficient $\varepsilon(\lambda)$ of gold nanoparticles dispersion was determined by the method reported in literature[2], by the following equation:

$$\ln(\varepsilon) = k ln(d) + a$$

with d core diameter of nanoparticles, and k and a dimensionless parameters ($k = 3{,}32111$ and $a = 10{,}80505$). The arithmetic mean of the sizes obtained by optical and scattering analyses was selected, leading to a $\varepsilon(\lambda)$ of $2.0 \cdot 10^{8}$ $M^{-1}cm^{-1}$. The final concentration of the citrated AuNPs is therefore ~$6.3 \cdot 10^{-9}$ M.

Supplementary Characterization of Liposomes

Dynamic Light Scattering and Zeta-Potential

	D_h (nm)	Zeta P
DOPC	118,6 ± 0.2	-12 ± 2
DPPC	178,7 ± 0.1	-10 ± 2

Table S3 *Hydrodynamic diameter obtained from Dynamic Light Scattering and Zeta Potential values of synthetic liposomes.*

Evaluation of Liposomes concentration

The lipid concentration in the starting colloidal dispersion was estimated to be 4 mg/mL from the initial lipid and water amounts employed in the formation and swelling of lipid films, assuming the absence of lipid loss due to the extrusion procedure. The liposomes concentration in the final dispersion was subsequently calculated considering the hydrodynamic diameter of each liposomal batch (Table S3 of SI). In particular, the liposomal surface area (surface area=$4\pi r^2$) can be calculated from the liposome diameters; the doubled surface can be subsequently divided by the lipid cross section (0,5 nm^2) in order to obtain the lipid number per liposome, assuming that approximately one half of the lipids is localized in the external leaflet of a liposome, since the bilayer thickness, about 4-5 nm, is negligible with respect to the liposomes' average diameter. Eventually, the total weighted lipid concentration was divided by the total number of lipids per liposome, yielding the real liposome concentration, which is reported in Table S4 for each liposomes' dispersion.

	Concentration (M)
DOPC	$3,2 \cdot 10^{-8}$
DPPC	$2,1 \cdot 10^{-8}$

Table S4 Final liposomes' concentration in each liposomal batch.

The liposomal dispersions were diluted to reach a final concentration of $1,2\cdot10^{-8}$ M before use.

Small Angle X-Ray Scattering

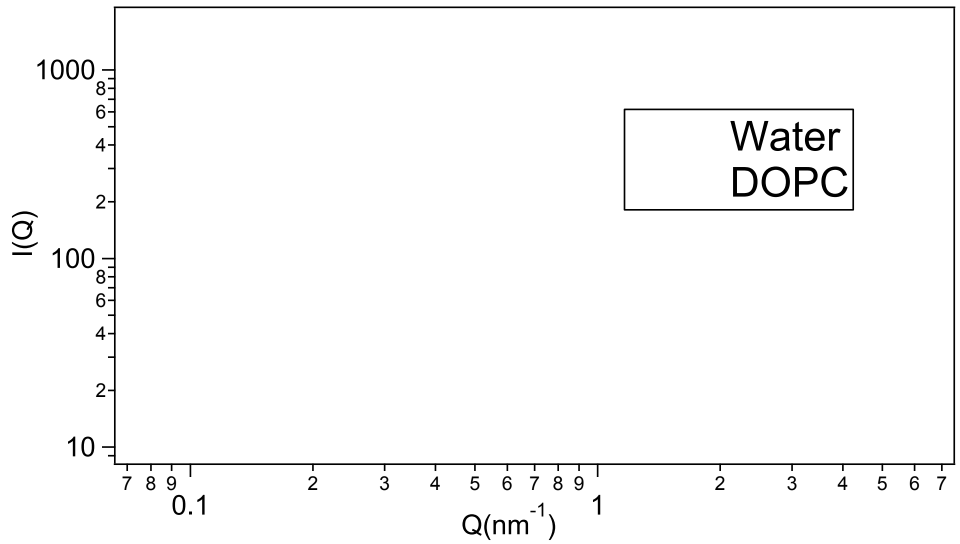

Figure S4 *Comparison between the scattering intensity of water and the scattering intensity of 10 µL of liposome dispersions (12 nM) in 300 µL of water.*

Supplementary Characterization of liposomes/AuNPs hybrids

Preparation of liposomes/AuNPs hybrids

The hybrid samples were prepared as follow: 10 µL of liposome dispersions (12 nM) were incubated with 300 µL of AuNPs 6,3 nM, in order to have a

liposomes/AuNPs number ratio of ~1/16. This liposomes/AuNPs number ratio was selected on the basis of our previous publication[3,4] which highlights that the aggregation of AuNPs on zwitterionic vesicles is promoted by low liposome amounts within the mix.

Cryo-TEM

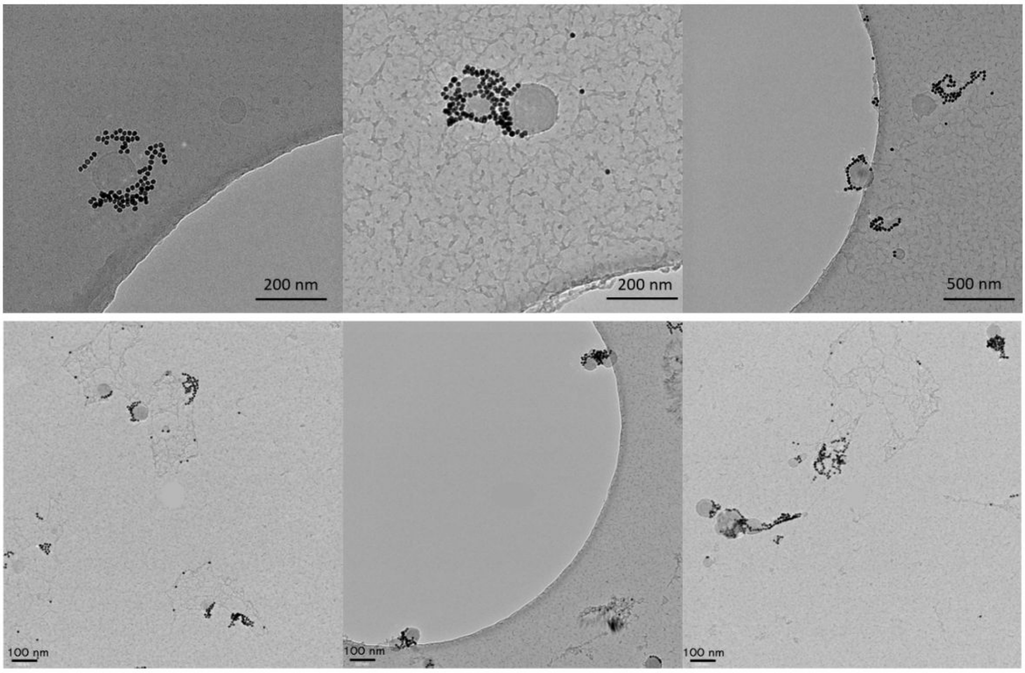

Figure S5 *Further examples of cryo-TEM images of AuNPs-DOPC hybrids.*

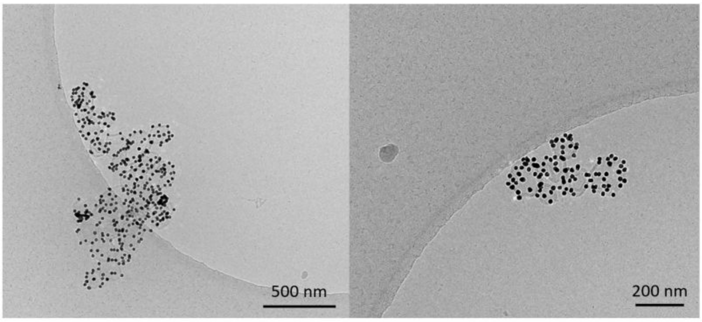

Figure S6 *Further examples of cryo-TEM images of AuNPs-DPPC hybrids.*

MD Simulations

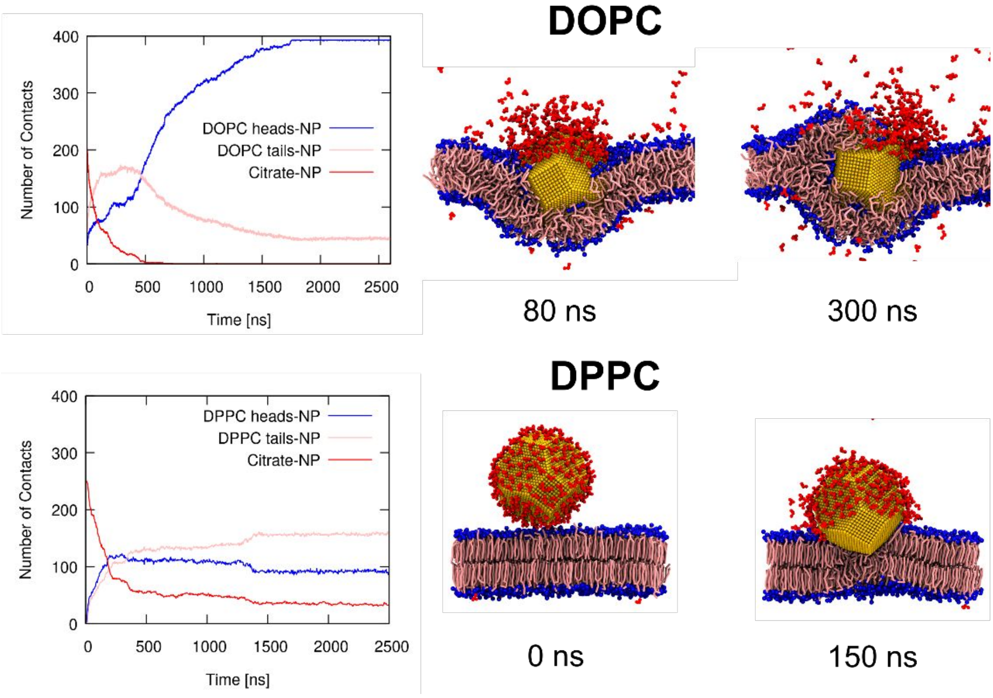

Figure S7: *Difference between the penetration process of a citrate 8 nm AuNP in DOPC and DPPC. For each kind of bilayer, we show the time evolution of the number of contacts between the NP and citrate molecules (in red), lipid headgroups (blue) and lipid tails (pink), and three snapshots from the corresponding simulations; in the snapshots, the NP is represented in yellow, the citrate in red, the lipid headgroups in blue and the lipid tails in pink. Time t=0 corresponds to the first NP-membrane contact. The contact time series is shown only up to 2,5 μs; afterwards, the values are stable.*

Small-Angle X-Ray Scattering

To characterize the NPs- vesicles hybrids' formation, 10 μL of 12 nM DOPC or DPPC liposomal dispersions were challenged with 300 μL of 6.3 nM citrate-stabilized AuNPs. In order to gain information on the kinetic of AuNPs

aggregation, the SAXS profiles have been collected after 1s, 30s, 5m and 10 minutes of the incubation.

In the low q region, plotting $\log_{10}(I(q))$ vs $\log_{10}(q)$ it's possible to obtain the fractal dimension of the aggregates by the slope of the scattering profile[5], according to:

$$log_{10}(I(q) - B) = -p \; log_{10}(q)$$

Where B is the background and p the Porod exponent. Generally, p=1 represents the fractal dimension of a linear aggregate and p=2 represents the fractal dimension of a 2D object.[5]

The SAXS profiles of DOPC liposomes/AuNPs and DPPC liposomes/AuNPs in Figure 3 were fitted through a linear fit in the 0,1-0,3 nm^{-1} q-range, to obtain the slope values reported in table S5.

**Incubation time**	**AuNPs-DOPC**	**AuNPs-DPPC**
**1s**	-1,32 ± 0.02	-0,53 ± 0.03
**30s**	-1,90 ± 0.02	-0,94 ± 0.01
**5m**	-2,00 ± 0.02	-1,02 ± 0.01

**10m**	-2,01 ± 0.02	-1,06 ± 0.01

Tab S5: *Slopes of the linear fits of the SAXS profiles*;

The Structure factors for the scattering profiles reported in the insets of figure 4 (main text) were obtained as follow.

The scattering intensity (I(q)) is defined by the following equation:

$$I(q) = KN_pV_p^2(\Delta\rho)^2P(q)S(q) + B$$

With k instrumental constant, N_p scattering nanoparticles' number per unit volume, V_p nanoparticle's volume, $\Delta\rho$ contrast of the experiment, B background intensity, P(q) e S(q) form and structure factors, respectively.

In order to obtain the structure factor of the liposome/AuNPs complex, we divided the scattering intensity of the liposomes/AuNPs hybrid by the scattering intensity of the neat AuNPs dispersion:

$$\frac{I(q)_{Hyb}}{I(q)_{NP}} \sim \frac{S(q)_{Hyb}P(q)_{Hyb}}{S(q)_{NP}P(q)_{NP}}$$

For a diluted AuNPs dispersion the structure factor can be considered equal to 1. In addition, in the high-q region (0,1-1,6 nm^{-1}), the form factor of liposomes/AuNP hybrids can be approximated to the one of neat AuNPs, leading to the following equation:

$$\frac{I(q)_{Hyb}}{I(q)_{NP}} = S(q)_{Hyb}$$

The mean interparticle distance between the AuNPs within the aggregates (d) can be obtained from the S(q) vs q (nm^{-1}) plot (see inset of Figure 2b of the main text), by the following equation:

$$d = \frac{2\pi}{qmax}$$

With q_{max} value corresponding to the maximum of the correlation peaks reported in the insets of figure 4 (main text).

Agarose gel electrophoresis

In order to perform gel electrophoresis, the AuNPs and DOPC or DPPC dispersions were mixed as previously reported. The AuNPs-DOPC+DPPC sample were prepared as follow: 300 μL of 6,3 nM AuNPs aqueous solution were added to the mixture of 5 μL of 12 nM DOPC and 5 μL of 12 nM DPPC dispersions. The AuNPs-DOPC + AuNPs-DPPC sample was prepared mixing AuNPs-DOPC and AuNPs-DPPC hybrids after their formation. 15 μL of each sample were a mixed with 5 μL of glycerol in order to improve their deposition at the bottom of the wells.

Bibliography

1 W. Haiss, N. T. K. Thanh, J. Aveyard and D. G. Fernig, 2007, **79**, 4215–4221.

2 X. Liu, M. Atwater, J. Wang and Q. Huo, 2007, **58**, 3–7.

3 L. Caselli, A. Ridolfi, J. Cardellini, L. Sharpnack, L. Paolini, M. Brucale, F. Valle, C. Montis, P. Bergese and D. Berti, *Nanoscale Horizons*, , DOI:10.1039/d1nh00012h.

4 C. Montis, L. Caselli, F. Valle, A. Zendrini, F. Carlà, R. Schweins, M. Maccarini, P. Bergese and D. Berti, *J. Colloid Interface Sci.*, 2020, **573**, 204–214.

5 T. A. Ezquerra, M. Garcia-Gutierrez, A. Nogales and M. Gomez, *Applications of synchrotron light to scattering and diffraction in materials and life sciences*, 2009.
